# Breast self-examination prevalence and determinants in low- and middle-income countries: an umbrella review of systematic reviews and meta-analyses

**DOI:** 10.3389/fgwh.2026.1439187

**Published:** 2026-04-01

**Authors:** Befkad Derese Tilahun, Mulat Ayele, Biruk Beletew Abate, Tegene Atamenta Kitaw, Eyob Shitie Lake, Alemu Birara Zemariam, Gizachew Yilak

**Affiliations:** 1 Department of Nursing, College of Health Science, Woldia University, Woldia, Ethiopia; 2 Department of Midwifery, College of Health Science, Woldia University, Woldia, Ethiopia

**Keywords:** breast self-examination, determinants, low and middle-income countries, umbrella review, women

## Abstract

**Background:**

Low- and middle-income countries (LMICs) face a dual burden of infectious and chronic diseases, including breast cancer. Breast self-examination (BSE) is a vital tool for early detection, improving outcomes, and reducing mortality. Assessing its prevalence and determinants in LMICs is crucial for enhancing early diagnosis and treatment.

**Methods:**

A comprehensive search was conducted across PubMed, Web of Science, Scopus, Cochrane, and Google Scholar using PICO criteria to identify systematic reviews and meta-analyses on breast self-examination in Low- and middle-income countries. Methodological quality was assessed using the AMSTAR tool. A random-effects meta-analysis pooled estimates, with heterogeneity (*I*^2^) and publication bias (funnel plot) evaluated. Forest plots presented pooled prevalence with 95% confidence intervals (CI).

**Results:**

This umbrella review included 10 studies (110,622 participants). The pooled BSE prevalence was 32.15% (confidence interval: 22.61–40.75), with high heterogeneity (*I*^2^ = 100%, *p* = <0.001). Significant determinants included BSE knowledge (OR = 3.95; 95% CI: 3.02–4.87), a positive attitude (OR = 2.73; 95% CI: 2.02–3.45), and a family history of breast cancer (OR = 1.81; 95% CI: 1.23–2.38).

**Conclusion:**

The prevalence of breast self-examination (BSE) in low and middle-income countries (LMICs) remains relatively low at 32.15%. Key factors influencing BSE practice include knowledge, attitudes, and family history of breast cancer. To enhance BSE practice, targeted strategies such as public awareness campaigns, training for healthcare providers, and incorporating family history assessments are essential. Additionally, strengthening research and policy initiatives can help address existing gaps in awareness, promote early detection, and improve breast cancer outcomes in low and middle-income countries.

**Systematic Review Registration:**

PROSPERO CRD42023491634.

## Introduction

Cancer is projected to become the leading cause of death globally in the coming decades, posing a significant challenge to global efforts aimed at increasing life expectancy (1). Among all cancers, breast cancer is the most commonly diagnosed in women, with an estimated 1.7 million new cases and 521,900 deaths globally (1). In 2017 alone, approximately 252,710 new cases of invasive breast cancer were diagnosed in women, with an estimated 40,610 deaths in the United States (2). Among women, it was the most prevalent form of cancer by a significant margin. Globally, breast cancer accounts for about 25.2% of all new cancer cases in women (3).

While breast cancer affects women in both developed and developing countries, the burden is increasingly shifting toward low- and middle-income countries (LMICs), where healthcare systems face resource constraints (4). By 2030, it was estimated that 70% of all breast cancer cases would occur in LMICs (5). In these regions, the incidence is rising across all age groups, with the highest increase observed in women under 50 (6). For example, in sub-Saharan Africa, breast cancer incidence varies from 19.3 to 38.1 per 100,000 women annually, with notable differences across regions such as Eastern and Southern Africa (7).

Early detection plays a critical role in improving survival outcomes. In high-income countries, over 70% of breast cancer cases are diagnosed at early stages (I and II), leading to improved prognosis and higher survival rates. In contrast, due to limited access to screening services like mammography, only 20%–60% of cases in LMICs are detected early (8, 9). As a result, women in LMICs often present with advanced-stage disease, which is associated with reduced survival and increased mortality (10–12).

In these low-resource settings, breast self-examination (BSE) is a particularly important early detection method. While not a replacement for clinical screening, BSE is a low-cost, accessible practice that can increase awareness, promote early presentation, and empower women (13). Studies from high-income countries suggest that routine breast self-examination (BSE) does not significantly reduce breast cancer mortality and may lead to false positives and unnecessary biopsies, causing psychological distress (14, 15). However, in low- and middle-income countries (LMICs), where access to mammography is limited, BSE often remains the most feasible early detection method. Its role, whether as a substitute or a complementary strategy, remains unclear in policy and practice. For BSE to be effective in LMICs, its function must be clearly defined within the context of local resources, health systems, and cultural norms (16, 17).

Studies have shown that a variety of factors influence BSE practice, including knowledge of breast cancer (18–24), attitudes toward BSE (18–20, 22), family history of breast cancer (19, 23, 25), and educational level (21, 23, 25, 26).

Despite recommendations by organizations such as the American Cancer Society, which encourages education on BSE from age 20 onwards (27). The practices of BSE in LMICs remain inconsistent. Reported prevalence rates vary widely across studies, with estimates ranging from 11.23% (28), 20.43% (23), 56.31% (29), and 43.14% (30). This inconsistency presents challenges for designing effective awareness and screening programs. Although multiple systematic reviews have addressed BSE in LMICs, no single synthesis has unified their findings. Therefore, this umbrella review aims to:

1. Provide a comprehensive estimate of the prevalence of BSE, and
2. Summarize evidence regarding the determinants of BSE practice in LMICs.

By synthesizing existing reviews, this study offers a broader perspective to support public health initiatives for early detection and breast cancer control in resource-limited settings.

## Methods

### Study design and protocol

This umbrella review utilized the approach outlined in the umbrella review of systematic review and meta-analysis (SRMA) research, along with the meta-analysis of observational studies (MOOSE) methodology (23, 28, 30–36). These methodologies involve detailed checklists comprising 35 items that offer guidance for conducting and reporting observational studies that are susceptible to significant bias and confounding, particularly when analyzing retrospective data (Supplementary File S1). This umbrella review was registered with the Prospective International Register of Systematic Reviews (PROSPERO, number CRD42023491634) and was conducted following the guidelines outlined in the Preferred Reporting Items for Systematic Review and Meta-Analyses (PRISMA). The review involved a methodical examination of the studies included, which encompassed systematic reviews and meta-analyses, with a specific focus on the occurrence of breast self-examination and the factors that influence it in low- and middle-income countries.

### Search strategy

A comprehensive literature search was conducted to identify studies on the prevalence and determinants of breast self-examination (BSE) in low- and middle-income countries (LMICs). The search spanned multiple electronic databases, including PubMed, Web of Science, the Cochrane Database of Systematic Reviews, Scopus, International Scientific Indexing (ISI), and Google Scholar. The PICO framework guided the development of the search strategy, utilizing a combination of keywords and Medical Subject Headings (MeSH) related to prevalence (e.g., “proportion,” “incidence,” “epidemiology,” “determinants,” “factors”), BSE practices (e.g., “breast self-examination,” “breast self-examination practices”), population (e.g., “women,” “low- and middle-income countries”), and study design (e.g., “systematic review,” “meta-analysis,” “review”). Boolean operators (AND, OR) were used to structure and refine the search.

To enhance comprehensiveness, grey literature was included through manual searches of relevant organizational websites, conference proceedings, and institutional repositories. Google Scholar served as an additional source for identifying potentially relevant but unindexed or unpublished studies. A snowballing technique was also employed, whereby reference lists of selected articles were reviewed to identify further eligible studies.

The literature search was conducted between December 26 and 30, 2024, and included all articles published up to the date of the search. Two independent reviewers (BDT and MA) carried out the screening and selection process. Discrepancies were resolved through discussion, and no conflicts arose (Supplementary File S2).

#### Population (P)

Women residing in low- and middle-income countries (LMICs), as classified according to the World Bank and International Monetary Fund income groupings.

#### Intervention/phenomenon of interest (I)

Breast self-examination (BSE) practices, defined as a woman examining her own breasts at home by inspection and palpation to identify possible lumps, distortions, or swelling as a preventive measure for breast cancer detection. Women who reported ever performing BSE, either regularly or irregularly, were considered to have practiced BSE.

#### Comparator (C)

Women who did not perform breast self-examination.

#### Outcome (O)

The primary outcomes were (i) the magnitude (prevalence) of breast self-examination practices and (ii) factors associated with breast self-examination practices among women in LMICs.

### Eligibility criteria

#### Inclusion criteria

This review of reviews included SRMAs that fulfilled the following criteria: (1) included a clearly defined literature search strategy, (2) evaluated the included studies using an appropriate tool, and (3) followed a standardized approach for combining studies and presenting summary estimates.

#### Exclusion criteria

Studies were excluded from consideration for this study based on any of the following grounds: (1) absence of relevant measures of interest, (2) language other than English, and (3) inclusion of narrative reviews, editorials, correspondence, abstracts, and methodological studies.

### Identification and study selection

All the identified studies were imported into the EndNote X8 reference manager software, and any duplicate articles were removed. The screening process involved evaluating the titles and abstracts of the studies. Three authors (BD, ES, and MA) together screened and assessed the articles. The full text of the selected studies was then evaluated based on their objectives, methodology, participants/population, and key findings related to the prevalence of breast self-examination practice and its determinants among women in low- and middle-income countries. In case of any disagreements during the screening process, a consensus meeting was held involving other senior reviewers (TA and BB) to resolve them.

### Data extraction

Data from the studies included were collected using a standardized data abstraction form created in Excel. The following information was extracted for each study: (1) identification details (last name of the first author and publication year), (2) magnitude measurement (prevalence of breast self-examination practices), (3) factors associated with breast self-examination practices (odds ratio or relative risk) along with their 95% confidence intervals, (4) number of studies included, (5) total number of samples included, (6) methods and scores for assessing publication bias, (7) methods and scores for assessing study quality, (8) methods of data synthesis (random or fixed-effects model), and (9) the primary conclusion of the study.

### Quality assessment

The studies’ quality was evaluated by utilizing the Assessment of Multiple Systematic Reviews (AMSTAR) checklist to assign scores (37). AMSTAR, short for A Measurement Tool to Assess Systematic Reviews, functions as a tool for appraising the methodological quality of systematic reviews. It consists of 11 criteria that scrutinize various elements like the research question, study selection and data extraction, and the methods employed to combine study results. Each criterion is evaluated as “Yes,” “No,” “Can't Answer,” or “Not Applicable,” with the total score reflecting the overall quality of the systematic review. The total AMSTAR scores (ranging from 0 to 11), which is also categorized as high quality (score ≥8), medium quality (score 4–7), or low quality (score ≤3). Three authors (BD, MA, and GY) evaluated the quality of each study, considering factors such as methodological quality, sample selection, sample size, comparability, outcome, and statistical analysis. In the event of disagreement among the three authors, two additional authors (ESL and AA) were consulted to discuss and resolve the disagreement (Table 1).

**Table 1:** Methodological quality of the included studies about breast self-examination practice among women in low- and middle-income countries based on the AMSTAR tool, 2024.

Authors (year)	Q1	Q2	Q3	Q4	Q5	Q6	Q7	Q8	Q9	Q10	Q11	Total
Yeshitila et al. (2021) (23)	Yes	Yes	Yes	Yes	Yes	Yes	Yes	Yes	Yes	Yes	No	10
Kassie et al. (2021) (28)	Yes	Yes	Yes	Yes	Yes	No	Yes	No	Yes	Yes	Yes	9
Mekonnen (2020) (29)	Yes	Yes	Yes	Yes	Yes	Yes	Yes	Yes	Yes	Yes	No	10
Seifu (2021) (33)	Yes	Yes	Yes	Yes	Yes	Yes	Yes	Yes	Yes	Yes	Yes	11
Halim (2023) (30)	Yes	Yes	No	No	Yes	Yes	Yes	Yes	Yes	Yes	Yes	9
Pal et al. (2021) (35)	Yes	Yes	Yes	No	Yes	Yes	Yes	Yes	Yes	Yes	Yes	10
Ahadinezhad et al. (2023) (31)	Yes	Yes	Yes	Yes	Yes	Yes	Yes	No	Yes	Yes	Yes	10
Samuel et al. (2022) (36)	Yes	Yes	Yes	Yes	Yes	Yes	Yes	No	Yes	Yes	Yes	10
Badakhsh et al. (2018) (32)	Yes	Yes	Yes	No	Yes	Yes	Yes	Yes	Yes	Yes	No	9
Gupta et al. (2019) (34)	Yes	Yes	Yes	Yes	Yes	Yes	Yes	Yes	No	Yes	Yes	10

AMSTAR, assessment of multiple systematic reviews.

Q1: *a priori* design; Q2: Duplicate study selection and data extraction; Q3: Search comprehensiveness; Q4: Inclusion of grey literature; Q5: Included and excluded studies provided; Q6: Characteristics of the included studies provided; Q7: Scientific quality of the primary studies assessed and documented; Q8: Scientific quality of included studies used appropriately in formulating conclusions; Q9: Appropriateness of methods used to combine studies’ findings; Q10: Likelihood of publication bias was assessed; Q11: Conflict of interest – potential sources of support were clearly acknowledged in both the systematic review and the included studies.

### Data processing and analysis

The selection of the meta-analysis model was influenced by the level of heterogeneity observed among the studies, which was evaluated using Higgins’ *I*^2^ statistics. Based on Higgins's *I*^2^ values below 49%, between 50% and 75%, and above 75% indicate low, moderate, and high levels of heterogeneity, respectively. We intended to pool the estimates with fixed-effects models if the level of heterogeneity was <50%. However, there was a high level of between-studies heterogeneity. Thus, the pooled prevalence estimates were calculated with the random-effects model, which accounts for both within-study and between-study variations

## Results

### Literature search findings

**Figure 1:**
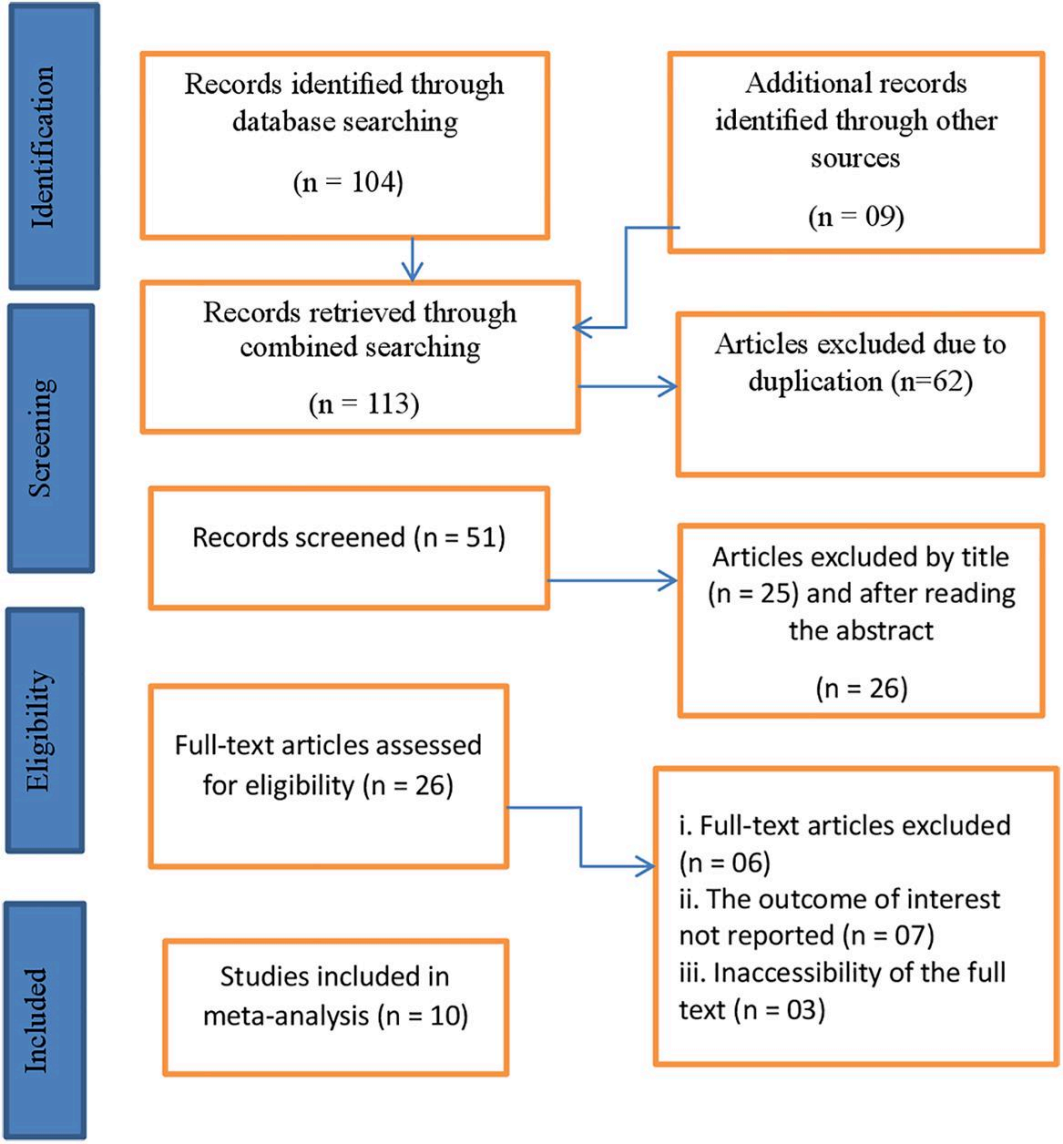
PRISMA flow diagram showing searching strategies, for breast self-examination practice among women in low- and middle-income countries, 2024.

The first database search discovered 113 articles. After removing duplicates, there were 51 distinct articles. Following the screening of titles and abstracts, 23 articles were excluded based on titles and 13 based on abstracts. The remaining articles were subject to a detailed full-text evaluation to determine their eligibility for inclusion. 6 studies were excluded due to differing outcome estimates, 7 because the outcome of interest was not reported, and an additional 3 papers were excluded due to the inaccessibility of the full text. As a result, a total of 10 studies (23, 28, 30–36) were included in the present umbrella review (Figure 1).

### Characteristics of the included review studies

This umbrella review comprised ten systematic reviews and meta-analyses (23, 28, 30–36), which collectively included a total of 310 primary cross-sectional studies involving a sample size of 110,622 women. The number of primary studies per systematic review and meta-analysis (SRMA) varied, ranging from 12 (29) to 56 (33). Similarly, the sample size per SRMA ranged from 4,129 (29) to 19,228 (33) women. All included SRMAs examined the prevalence and factors associated with breast self-examination practice in low- and middle-income countries. Out of the included reviews, five were conducted in Ethiopia (23, 28, 29, 33, 36), two of them from India (34, 35), two from Iran (31, 32), and one from Indonesia (30). Based on the included SRMA, the prevalence of breast self-examination practice ranged from 11.23% (95% CI: 7.67, 14.78), *I*^2^ = 97.4% (28) to 56.31% (95% CI: 44.37, 68.25), *I*^2^ = 98.5% (29) (Table 2).

**Table 2:** Characteristics of the included review studies on breast self-examination practice among women in low- and middle-income countries, 2024.

Authors (year)	Review objective	Search strategy	Population	Included studies	Sample size	Risk of bias	Reported prevalence	AMSTAR quality
Ahadinezhad et al. (2023) (31)	To estimate the pooled prevalence of breast self-examination practices in Iranian women	PubMed, Cochrane Library, Hinari, Google Scholar, CINAHL, and Global Health database. The search date was January 1, 2012, to September 11, 2022 clear searching term was defined. All available studies from January 1, 2012, to September 11, 2022 were included. Clear inclusion and exclusion criteria were defined.	Iranian women	38, all cross-sectional	9,960	The quality of included studies was appraised	15.46 (14.83–16.09), *I*^2^ = 98.4%	10
Badakhsh et al. (2018) (32)	To determine attitudes and practice regarding breast cancer early detection techniques [breast self-examination (BSE), clinical breast examination (CBE) and mammography] among Iranian woman.	International databases [MEDLINE (PubMed interface), Google Scholar and ISI Web of science (web of science interface)] and national databases [scientific information database (SID) and MAGIRAN], National key journal (Iranian Journal of Breast Diseases) databases were used. Had clear inclusion and exclusion criteria. Searching date not stated.	Iranian Women	21, all were cross sectional.	10,521	The quality of included studies was appraised clearly using NOS	27%	9
Yeshitila et al. (2021) (23)	To estimate the pooled prevalence of Breast self-examination practice and its determinants among women in Ethiopia	PUBMED, Cochrane Library, Google Scholar, CINAHL, African Journals Online, Dimensions and summon per country online databases were included. Clear searching terms were defined.	Women in Ethiopia	40, all were cross sectional	17,820	The JBI-MAStARI tool was used for critical appraisal.	36.72% (29.90–43.53) *I*^2^ = 99.3%	11
Gupta et al. (2019) (34)	To identify the Risk factors of breast cancer and breast self-examination in early detection among Indian women	MEDLINE/PUBMED, Cochrane Database of Systematic Reviews, Cumulative Index to Nursing and Allied Health (CINAHL), Google Scholar, and SCOPUS were included. Clear searching terms were defined. Searching date not stated. Had clear inclusion criteria and exclusion criteria	Women's health care professionals	Of the studies, 37 were community-based, 8 hospital-based, and 12 involved professionals, students, or teachers.	17, 585	The quality of included studies was appraised clearly using NOS	55.5%	10
Halim (2023) (30)	To determine the pooled prevalence Breast Self-Examination Practice and Its Determinants among Women in Indonesia	Cochrane Library, PubMed, Google Scholar, and SINTA (Indonesian Web of Science and Technology Index. Clear searching terms were defined. Published articles starting) from September 2017–2022. were included. Clear inclusion and exclusion criteria were defined.	Women in Indonesia	41, all were cross sectional.	6361	The quality of included studies was appraised clearly using NOS	43.14% (36.08–50.20) *I*^2^ = 100%.	8
Kassie et al. (2021) (28)	To determine the pooled prevalence Breast Self-Examination Practice and Its Determinants among Female University Students in Ethiopia	PUBMED, Cochrane Library, Google Scholar, CINAHL, African Journals Online, Dimensions and summon per country online databases were included. Clear searching terms were defined.	Female University Students in Ethiopia	16, all were cross sectional.	5,743	Joanna Briggs Institute Meta-Analysis of Statistics Assessment and Review Instrument (JBI-MAStARI) was applied for critical appraisal.	11.22 (7.67–14.78) *I*^2^ = 97.4%.	11
Mekonnen BD. (2020) (29)	To determine the pooled prevalence Breast self-examination practice and associated factors among female healthcare workers in Ethiopia	PubMed, Medline, EMBASE, Global Health, Google Scholar, CINAHL and Scopus from April 2, 2020 to April 24, 2020 were included. Clear inclusion and exclusion criteria were defined	Female healthcare workers in Ethiopia	12, all were cross sectional	4,129	The quality of included studies was appraised clearly using NOS	56.31% (44.37–68.25) *I*^2^ = 98.5%	10
Pal et al.(2021) (35)	Conducted to evaluate the knowledge, attitude, and practice of breast cancer and its screening among women in India.	Published from 2010 to April 2020, was conducted in electronic databases of PubMed and Google Scholar. Clear inclusion and exclusion criteria were defined	Women in India	15, all were cross-sectional	7,545	The quality of included studies was appraised using NOS		10
Samuel et al. (2022) (36)	To determine the pooled prevalence of Ethiopian women's breast cancer self-examination practices and associated factors	PubMed, Cochrane Library, Hinari, Google Scholar, CINAHL, and Global Health database were used. articles conducted in Ethiopia between 2011 and 2020 were included. Clear inclusion and exclusion criteria were defined	Ethiopian women	34, all were cross-sectional	14,908	The quality of included studies was appraised clearly using NOS	36% (28–43)	9
Seifu (2021) (33)	To determine the pooled prevalence of Breast self-examination practice among women in Africa	PubMed, EMBASE, Science Direct, HINARI, Google scholar, WHO Global Index Medicus and African Journal Online (AJOL) were searched to retrieve all available studies. All articles published up to June 30, 2020 were included. Searching date not stated. Clear inclusion and exclusion criteria were defined	women in Africa	56, all were cross sectional	19, 228	The JBI-MAStARI was used for critical appraisal.	17.9% (13.36–22.94) *I*^2^ = 98.23%	11

### Methodological quality of the included SRMA studies

The methodological quality assessment of the included systematic reviews and meta-analyses (SRMAs) was performed using the AMSTAR tool (37). The quality scores, which were rated on an 11-point scale, ranged from 8 to 9, with an average score of 9.6 (Table 1).

### Pooled prevalence of breast self-examination practice

**Figure 2:**
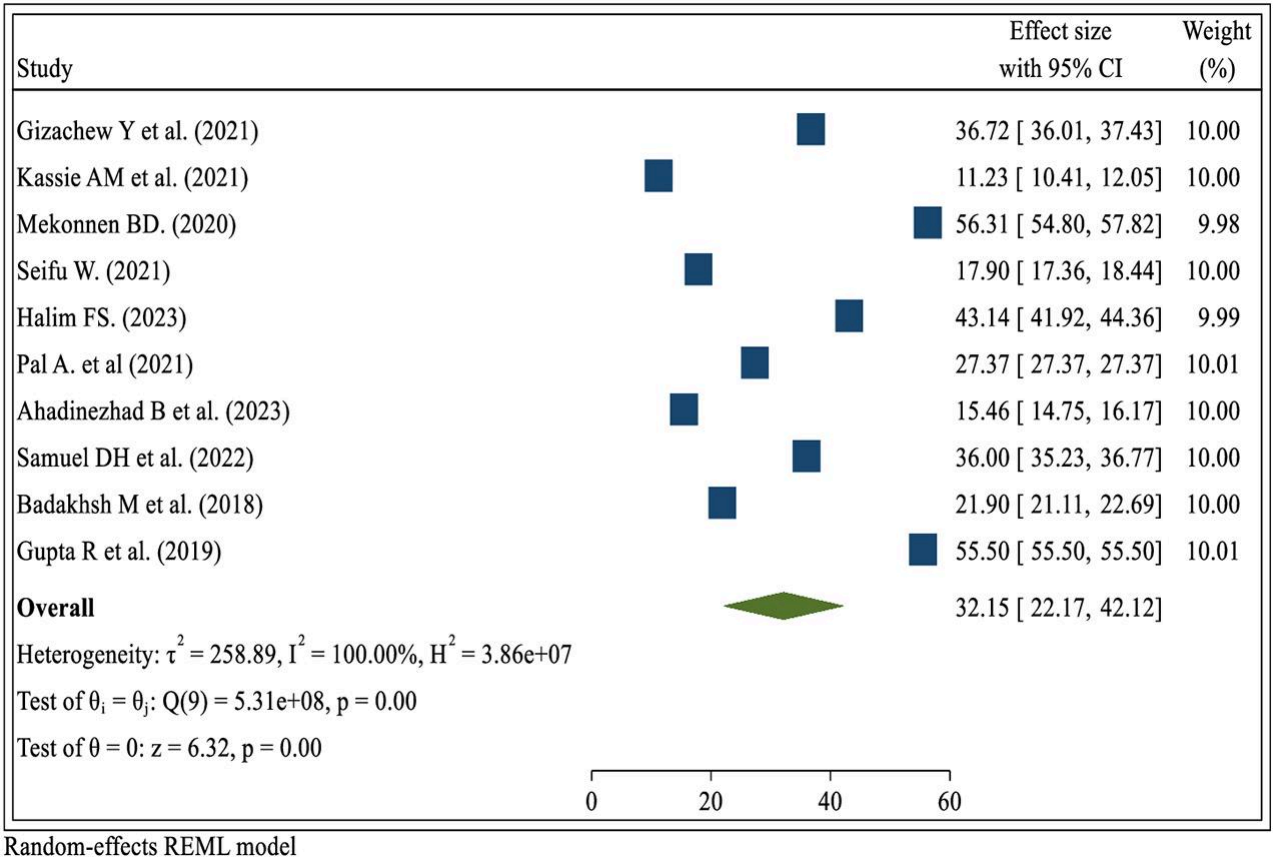
The pooled prevalence of breast self-examination practice among women in low- and middle-income countries, 2024.

The combined prevalence of breast self-examination practice among women in low- and middle-income countries was found to be 32.15% (CI: 22.17–42.12), indicating significant heterogeneity among the different reviews (*I*^2^ = 100.00%, *P* = 0.000), with an I^2^ value exceeding 75. Therefore, we used the random effect model to resolve the issue of heterogeneity among the included studies. Moreover, we considered subgroup analysis as a potential way of addressing heterogeneity. The findings are presented in the forest plot (Figure 2).

### Sensitivity analysis

**Figure 3:**
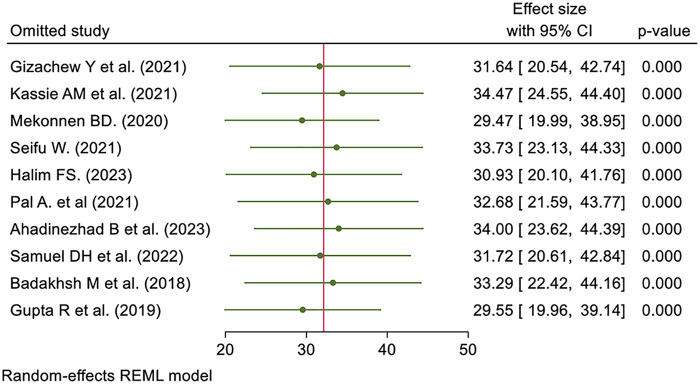
Sensitivity of pooled prevalence of breast self-examination practice among women in low- and middle-income countries, 2024.

We employed a leave-one-out sensitivity analysis to identify the potential source of heterogeneity in the analysis of the prevalence of breast self-examination practice. The results of this sensitivity analysis showed that our findings were not dependent on a single study. The pooled effect sizes remain statistically significant (*p* = 0.000) across all iterations, with effect sizes ranging narrowly from 29.47 to 34.47 and all 95% confidence intervals excluding zero. This consistency indicates that no individual study significantly alters the overall effect, confirming the stability and reliability of the meta-analytic findings (Figure 3).

### Publication bias

**Figure 4:**
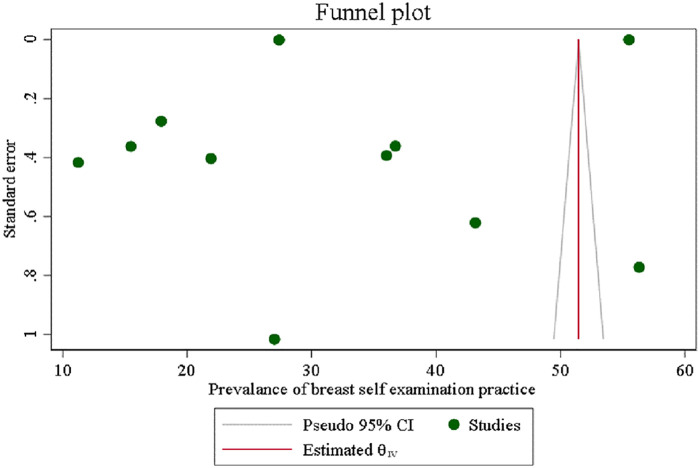
The funnel plot of breast self-examination practice among women in low- and middle-income counries, 2024.

In this umbrella review, an assessment of publication bias was conducted through the examination of a funnel plot, which visually analyzed the distribution of studies on breast self-examination for any signs of asymmetry. Furthermore, Egger's regression test was conducted to statistically assess the presence of publication bias, yielding a *p*-value of 0.589 (*p* > 0.05). A *p*-value above the commonly used significance threshold of 0.05 suggests that there is no significant evidence of publication bias in the included studies; this finding suggests the absence of publication bias (Figure 4).

#### Subgroup analysis

To address the considerable heterogeneity observed among the included meta-analyses and systematic reviews in this umbrella review, subgroup analysis was conducted by stratifying the data based on study year and participants’ profession.

#### Subgroup analysis by year of publication

**Figure 5:**
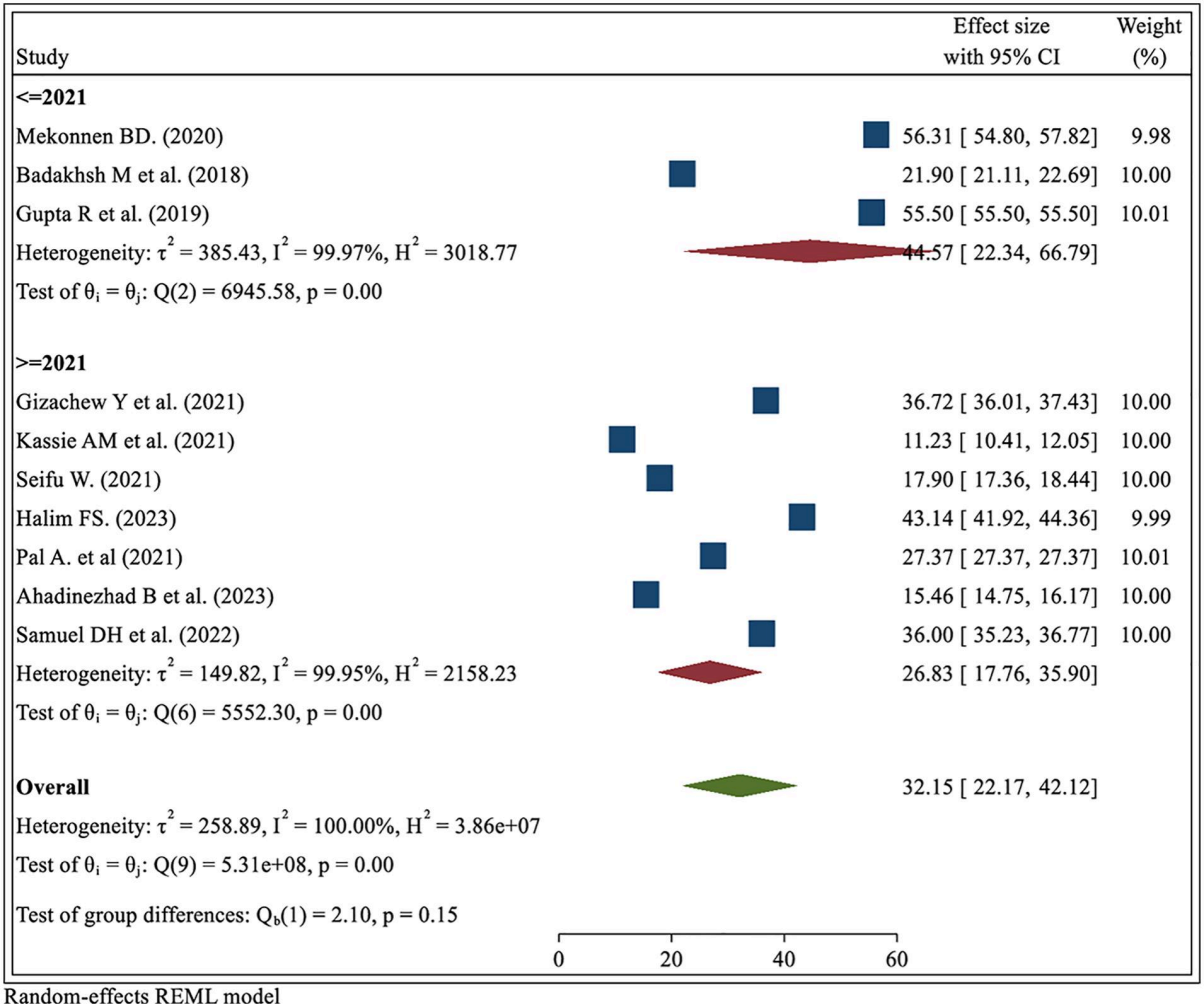
Subgroup analysis of breast self-examination practice by publication year among women in low- and middle-income countries, 2024.

The pooled prevalence of BSE practice in studies published on or before 2021 was higher (44.57%, 95% CI: 22.34–66.79) compared to studies published after 2021 (26.83%, 95% CI: 17.76–35.90). Despite the observed numerical difference, the test for group differences was not statistically significant (Q = 2.10, *p* = 0.15), indicating that the variation in BSE practice between the two time periods is not sufficient to confirm a temporal trend. High heterogeneity was observed within both subgroups (*I*^2^ > 99%), suggesting substantial variability among the included studies, likely due to differences in population characteristics, methodologies, or regional health contexts (Figure 5).

#### Subgroup analysis by participants’ profession

**Figure 6:**
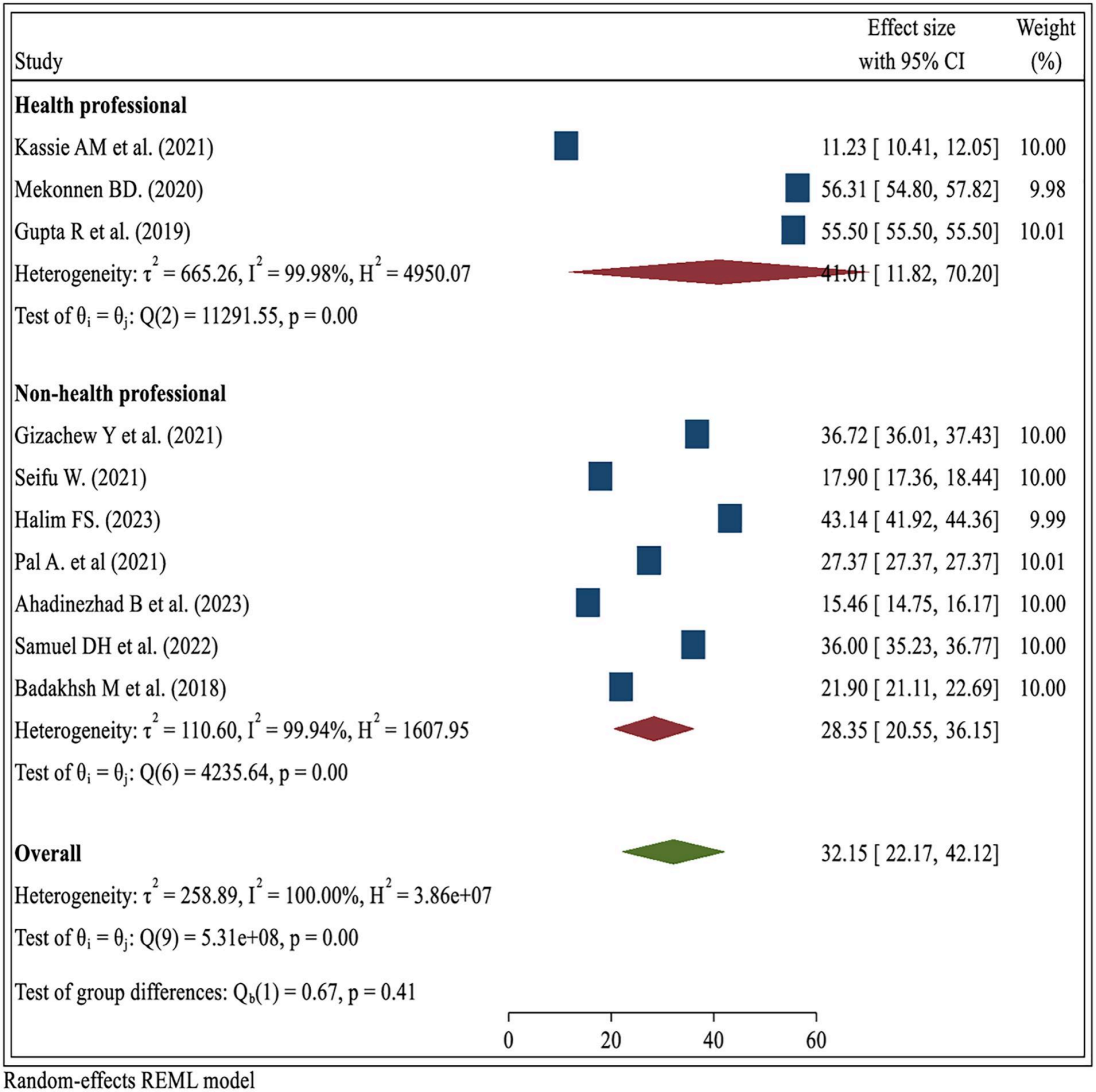
Subgroup analysis of breast self-examination practice by profession among women in low- and middle-income countries, 2024.

The pooled prevalence of BSE practice among health professionals was 41.01% (95% CI: 11.82–70.20), which is higher than that among non-health professionals, whose pooled prevalence was 28.35% (95% CI: 20.55–36.15). This difference suggests that professional background and likely associated health knowledge positively influence the likelihood of practicing BSE. Despite this numerical difference, the test for group differences was not statistically significant (Q = 0.67, *p* = 0.41), indicating that the observed variation between the two groups may be due to chance rather than a true effect. High heterogeneity was present in both subgroups (*I*^2^ > 99%), pointing to substantial variability among the studies, potentially influenced by differences in study populations, education levels, or regional health promotion efforts (Figure 6).

### Determinants of breast self-examination practice among women in low- and middle-income countries, 2024

#### Knowledge of breast self-examination practice

**Figure 7:**
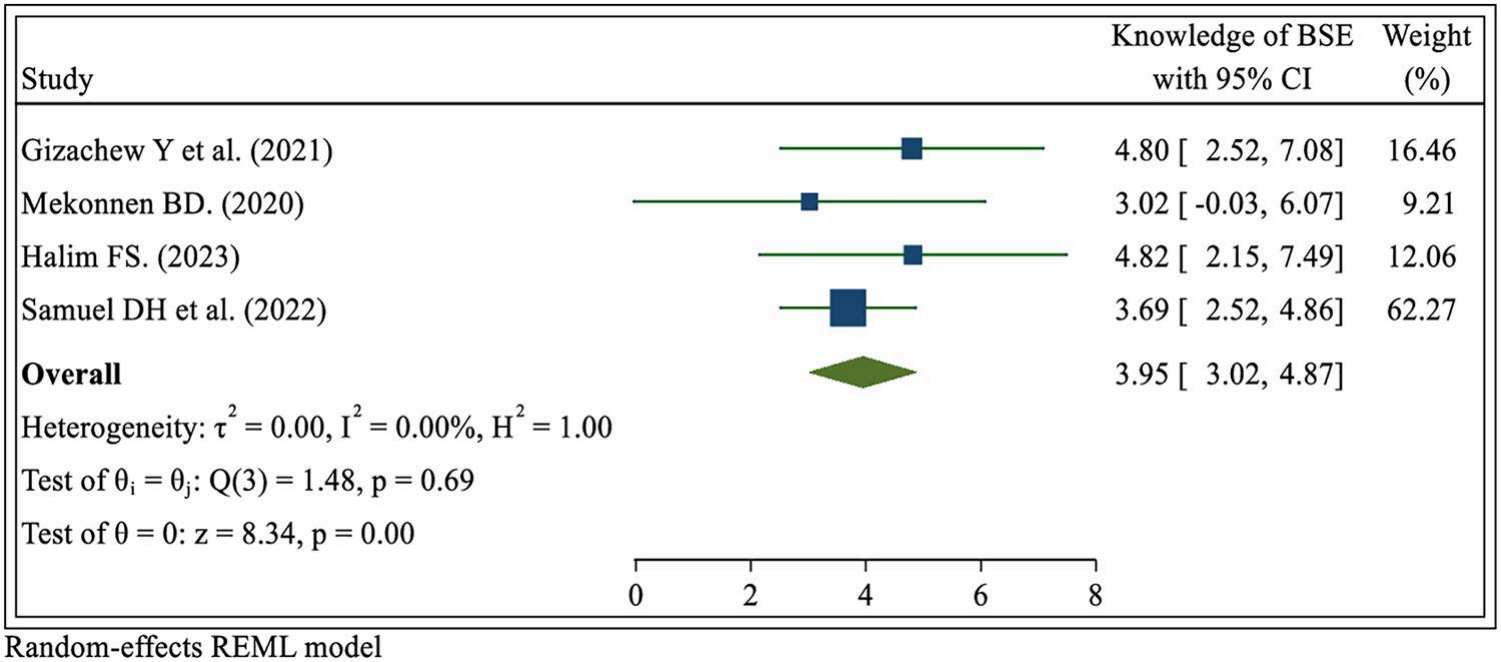
Umbrella review about the pooled effects knowledge of breast self-examination practice among women in low- and middle-income countries, 2024.

Four systematic review and meta-analysis studies (23, 29, 30, 36) have demonstrated a significant association between knowledge of breast self-examination practice and its prevalence. The findings indicate that women who knew breast self-examination were nearly four times more likely to engage in the practice compared to those who lacked such knowledge (OR = 3.95; 95% CI: 3.02, 4.87) (Figure 7).

#### Attitude for breast self-examination practice

**Figure 8:**
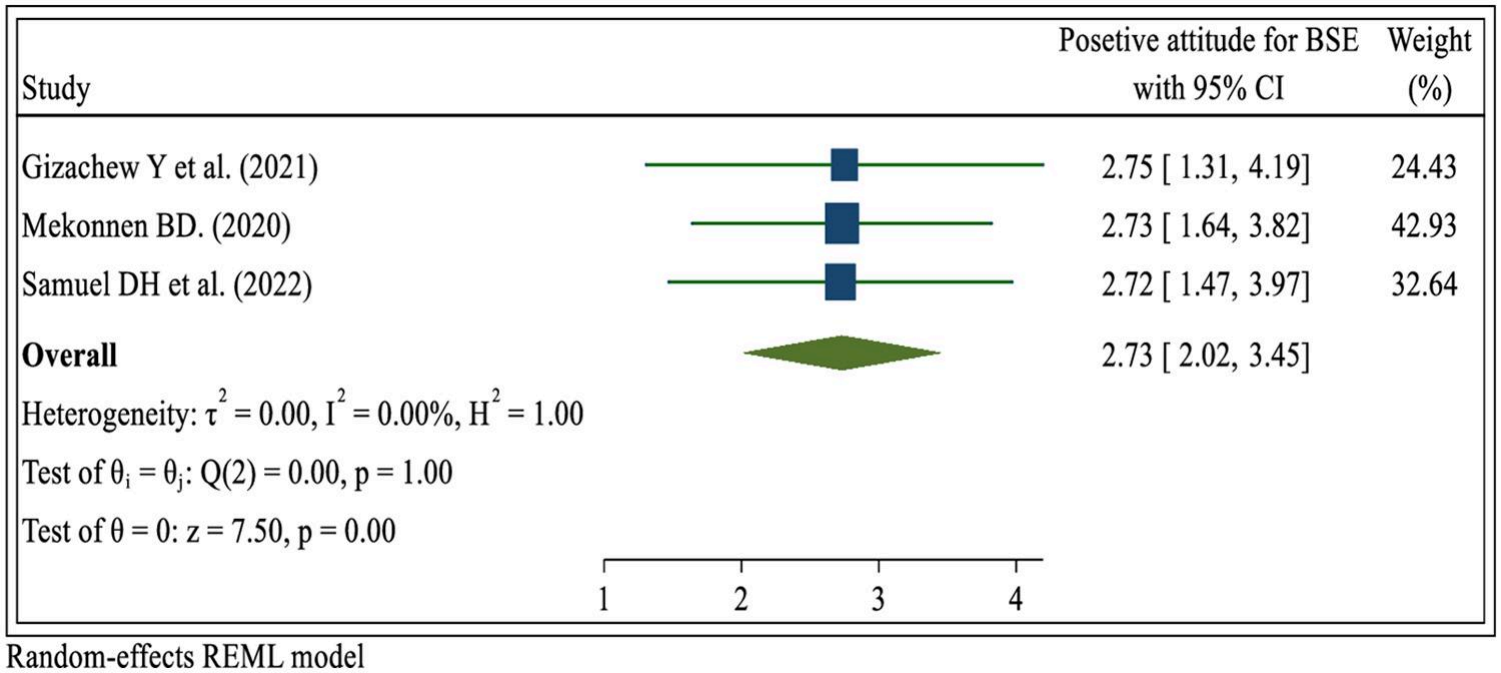
Umbrella review about the pooled effects of positive attitude for breast self-examination practice among women in low- and middle-income countries, 2024.

Three systematic review and meta-analysis studies (23, 29, 36). The study findings revealed a significant link between positive attitudes towards breast self-examination and the prevalence of breast self-examination. Women who held a positive attitude towards breast self-examination were nearly three times (OR = 2.73; 95% CI: 2.02, 3.45) more likely to engage in the practice compared to those who did not have a positive attitude towards breast self-examination (Figure 8).

#### Family history of breast cancer

**Figure 9:**
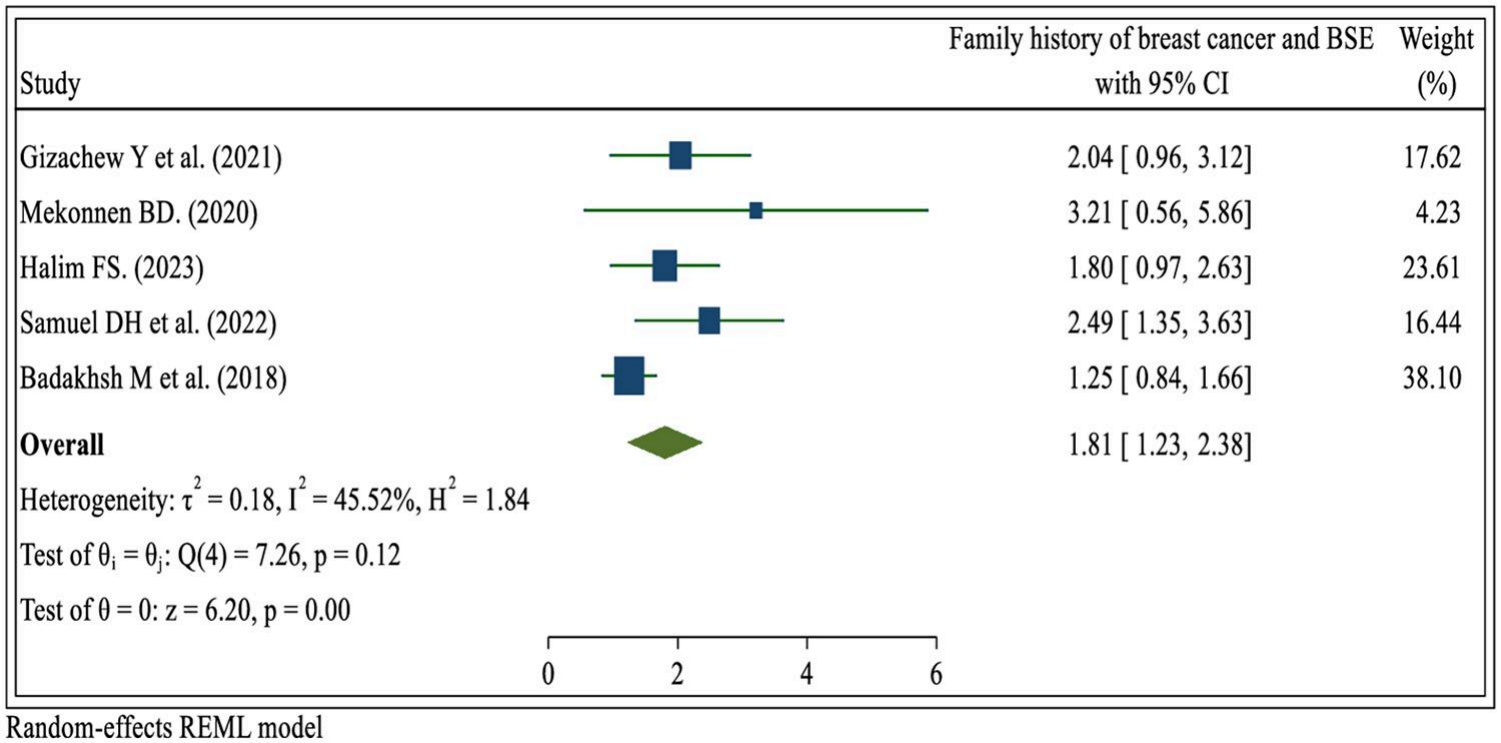
Umbrella review about the pooled effects of family history of breast cancer for breast self-examination practice among women in low- and middle-income countries, 2024.

Five systematic review and meta-analysis studies (23, 29, 30, 32, 36). According to the report, there was a significant association between a family history of breast cancer and the prevalence of breast self-examination practice. Women with a family history of breast cancer were nearly two times (OR = 1.81; 95% CI: 1.23, 2.38) more likely to engage in breast self-examination compared to those without a family history of breast cancer (Figure 9).

#### The educational status of the women

**Figure 10:**
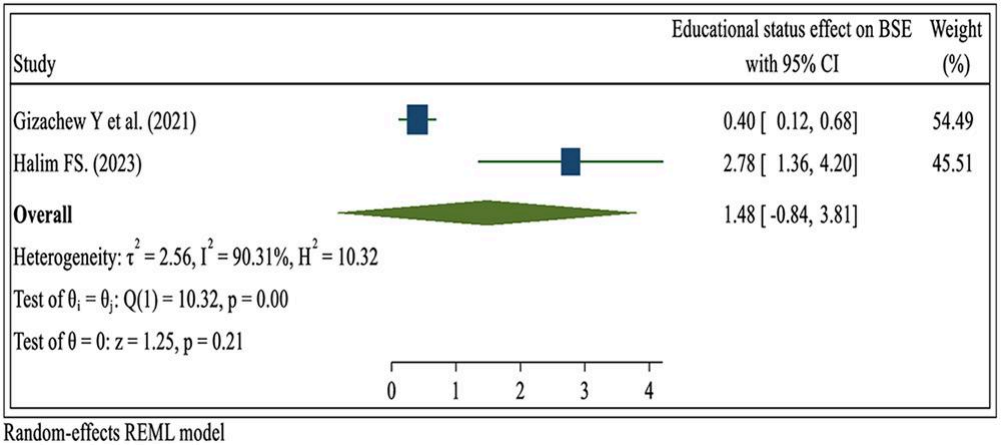
Umbrella review about the pooled effects of educational status for breast self- examination practice among women in low- and middle-income countries, 2024.

Two systematic review and meta-analysis studies (23, 30) reported that the education status of women did not have a significant association with the prevalence of breast self-examination practice (OR = 1.48; 95% CI: −0.84, 3.81) (Figure 10).

## Discussion

While systematic reviews and meta-analyses are considered high-level evidence for evidence-based healthcare practice, an umbrella review provides a more comprehensive and informative approach to clinical decision-making when multiple systematic reviews and meta-analyses have already been published on a specific research topic (38). Hence, this umbrella review aimed to provide a comprehensive summary of the available systematic reviews and meta-analyses on breast self-examination practices among women and their influencing factors in low- and middle-income countries. According to the World Bank's income classification, nations with a gross national income (GNI) per capita of **$1,005 or less** are categorized as *low-income*, while those with a GNI per capita ranging from **$1,006 to $3,955** fall into the *lower-middle-income* group. On this basis, **83 countries** are included in the classification, 2024/2025 (39).

In low- and middle-income countries, breast self-examination is considered a viable and practical approach for screening for breast cancer at an early stage. Early detection and screening are crucial for reducing breast cancer-related fatalities and serve as fundamental pillars of breast cancer control. While routine mammography screening may not be readily available in developing countries, it is essential to prioritize and promote breast self-examination. Breast self-examination is a straightforward, intuitively appealing, and non-invasive procedure that requires minimal time and incurs no medical costs (40).

In this umbrella review, the overall pooled prevalence of breast self-examination practice was 32.15% (95% CI: 22.61, 40.75). This finding was comparable to those of studies in Turkey (33.3%) (41). However, practice toward (early detection/screening methods) BSE was seen to be higher than studies, 27.37% in India (35), 21.9% in Iranian women, 15.46% (31) 43.6% among female secondary school teachers in Addis Ababa, Ethiopia (42), in Cameroon 15% (43), in Nepal Only 19.2% women had ever practiced BSE (44), Kuwait 21% (45), Eretria 11.7% (46). However, this finding was lower than what has been reported in studies in high-income countries among Eastern European immigrant women worldwide 44% (47), In Turkey, a systematic review of 78.5% (48) had a professional breast examination, in India 55.5% (34), 42.6% in Iraq (49), in Poland 56.1% (50), which could be attributed to the lack of awareness among many women in low- and middle-income countries regarding the significance of regular breast self-examinations for early detection of breast cancer. Limited access to health education programs, campaigns, and resources that offer information about breast health and self-examination techniques may contribute to this issue (51–53), and cultural beliefs and societal norms may influence healthcare practices in LMICs. In some cultures, discussing or examining one's own body, including the breasts, may be considered taboo or inappropriate (54). This can create barriers in low- and middle-income countries, where there are limited or no resources for population-based mammography screening. Therefore, health education programs, campaigns, and resources that offer information about breast health and self-examination techniques are essential for challenging this cultural taboo.

The practice of breast self-examination (BSE) was also compared by study publication year through subgroup analysis. A higher prevalence was reported in studies published on or before 2021 compared to those published after 2021. Although this difference was not statistically significant (Q = 2.10, *p* = 0.15), the observed trend may reflect the impact of global events such as the COVID−19 pandemic (55), which may have disrupted preventive health behaviors and reduced emphasis on self-care practices like BSE. High heterogeneity was observed within both subgroups (*I*^2^ > 99%), suggesting considerable variation across studies. This may be due to differences in study design, participant demographics, and contextual factors such as access to healthcare, cultural beliefs about breast health, and the level of public health investment in each country (56, 57). These findings highlight the importance of understanding the broader health system and sociocultural context influencing BSE practice and suggest a need for continued investment in context-specific health education programs that address these barriers.

Subgroup analysis revealed notable differences in breast self-examination (BSE) practice across population groups. The highest prevalence was observed among healthcare professionals compared to non-healthcare professionals (50, 58, 59), likely due to their medical training, greater exposure to breast health education, and routine engagement with clinical preventive practices. Conversely, the lowest prevalence was reported among non-health professionals (29, 45), underscoring the need for more robust community-level health promotion and advocacy efforts. These disparities in BSE practice are likely influenced by broader systemic and contextual factors, including differences in health infrastructure, public health investment, and access to accurate health information (10, 60). Additionally, cultural norms and societal perceptions play a significant role in shaping attitudes toward breast health, with stigmatization in some communities further limiting awareness and open dialogue around BSE (61, 62). Addressing these multifaceted barriers requires targeted, culturally sensitive, and linguistically appropriate interventions, particularly in low-resource settings, to promote early detection, improve understanding of breast cancer risk factors, and encourage regular BSE as a simple, cost-effective preventive strategy (62).

This umbrella review showed that good knowledge of breast cancer is significantly associated with the practice of breast self-examination. This is consistent with other studies (34, 44, 63–68). This may be explained by knowledge of breast cancer and BSE being considered essential precursors to women's adherence to practicing BSE. Women with higher knowledge scores regarding breast cancer and BSE were more likely to practice BSE than those with lower knowledge scores. Understanding the potential warning signs of breast cancer, such as a new lump or mass, nipple discharge, changes in breast shape or size, skin dimpling, or nipple inversion, may also increase breast self-examination practices (69–71). Thus, to increase women's adherence to BSE, more efforts are needed to improve their knowledge about this deadly disease and ways of prevention. Thus, it is vital to collaborate with healthcare providers to explore more effective ways to convey information on breast cancer screening to women, especially those who have not previously participated in any screening practice.

This umbrella review showed that women with positive attitudes increased breast self-examination practices. The results of this study are in line with the findings (45, 65, 72). This may be because a positive attitude can help motivate individuals to prioritize their health and make regular BSE a part of their self-care routine. When they have a positive outlook, they are more likely to take proactive steps toward maintaining their well-being, and a positive attitude fosters a sense of self-awareness and encourages individuals to remain informed about breast health and the importance of early detection. It helps create a mindset that values personal health and empowers individuals to take charge of their well-being (36). A positive attitude fosters motivation and a proactive mindset. It encourages individuals to take responsibility for their health and prioritizes regular BSE as part of their self-care routines. With a positive attitude, individuals are more likely to view BSE as an important aspect of maintaining their well-being.

In this umbrella review, a family history of breast problems was found to be a significant predictor of BSE practice. Studies reporting a family history of benign breast problems were more likely to perform BSE than those with no family history of breast problems. These findings are inconsistent with the findings (45, 63, 65, 66, 73). This might be because, when a family member is diagnosed with breast cancer, it raises awareness within the family about the disease and its potential risks. This heightened awareness can lead to a better understanding of the importance of early detection through regular breast self-examinations. Family members often share health information and experiences. If a family member has undergone regular breast self-examination or has been diagnosed with breast cancer at an early stage, they can share their knowledge and personal stories, emphasizing the importance of self-examination for early detection. This sharing of information can encourage other family members to adopt the practice (74). Women with these characteristics have more opportunities to visit health providers and receive recommendations about taking the test for diagnosis.

Education level is a factor that increases individuals’ awareness and self-confidence in their health. Therefore, an increase in education level plays an active role in increasing the awareness of breast cancer, knowing, and practicing BSE. Although recent reviews have reported that educational status is an important predictor of participation in breast cancer screening, this umbrella review showed that there was no significant association between educational level and BSE practice. This umbrella review agrees with the findings (44, 64, 75). This might be because while educational level can contribute to better awareness and understanding of BSE, it does not guarantee that individuals with higher education will always engage in regular BSE. Similarly, individuals with lower educational levels can still practice BSE effectively if they can access accurate information and resources (67, 68).

Cultural beliefs and practices, economic constraints affecting healthcare access, and social norms play crucial roles in shaping individuals’ behaviors towards preventive health measures like BSE. Studies have highlighted the significance of cultural perceptions, economic disparities, and social support networks in influencing BSE behaviors among women in LMICs (76–78). To develop effective interventions, future research should delve deeper into these multifaceted influences to create culturally sensitive and contextually relevant strategies promoting BSE practices in LMICs.

Addressing these barriers requires a multifaceted approach involving increased awareness through targeted health education campaigns, improved healthcare infrastructure, culturally sensitive interventions, and initiatives to enhance the affordability and accessibility of screening services. It is essential to empower women with the knowledge, resources, and support to promote regular breast self-examinations and facilitate the early detection of breast cancer in LMICs.

## Strengths and limitations of the study

We made extensive efforts to reduce the risk of bias by conducting thorough searches across multiple databases and involving two independent researchers who reached a consensus without any disagreement. To the best of our knowledge, no comprehensive assessment in the form of an umbrella review has been conducted on breast self-examination in low- and middle-income countries despite the availability of various empirical studies and specific systematic review and meta-analysis (SRMA) studies.

Although we have taken steps to minimize or address potential limitations, the absence of similar reviews presents a challenge when directly comparing our findings with those of other studies. As a result, we primarily relied on comparisons with individual primary studies to draw meaningful conclusions. In our study, noticeable heterogeneity was apparent, predominantly arising from the diverse study designs included in the meta-analysis and systematic review within our umbrella review. Lastly, the exclusion of non-English language studies, while common in systematic reviews, may have introduced language bias, potentially omitting relevant findings from regions where research is not typically published in English. This limitation is particularly relevant in the context of LMICs, where local-language publications may contain important data.

## Conclusion and recommendation

The results from the umbrella review, which compiled data from 10 studies involving a substantial sample of 110,622 participants, shed light on the prevalence of breast self-examination practices among women in low- and middle-income countries, indicating a rate of 31.68%. Notable associations were identified for factors such as knowledge, positive attitude, and family history of breast cancer. To comprehensively improve breast health outcomes in low- and middle-income countries (LMICs), a multifaceted and context-specific approach is essential. Educational campaigns should be culturally tailored and community-based, utilizing trusted local channels such as community health workers, radio programs, schools, and women's groups to enhance knowledge and foster positive attitudes toward breast self-examination (BSE). Training healthcare providers through short, practical workshops that emphasize both clinical and communication skills will equip them to effectively teach BSE techniques and encourage regular practice. To integrate family history assessments feasibly into clinical consultations, simple screening tools can be used during patient intake by trained community health workers or nurses, especially in rural settings where resources are limited. These tools can help identify high-risk individuals for targeted counseling and follow-up. Additionally, localized research should be supported to better understand the factors contributing to regional variations in BSE practices, with continuous monitoring and evaluation mechanisms in place to refine interventions over time. By implementing these comprehensive strategies, it is possible to enhance breast health awareness, promote early detection practices, and ultimately reduce the burden of breast cancer in low- and middle-income countries.

## Data Availability

The original contributions presented in the study are included in the article/Supplementary Material, further inquiries can be directed to the corresponding author.
