# Supplementary material for: Breast self-examination prevalence and determinants in low- and middle-income countries: an umbrella review of systematic reviews and meta-analyses
Source: Front Glob Womens Health. 2026 Apr 1;7:1439187. doi: 10.3389/fgwh.2026.1439187 (PMC13079669; doi:10.3389/fgwh.2026.1439187)
Supplement: Supplementary file 1 [file Supplementaryfile1.docx]

S 1 file; MOOSE checklist on breast self-examination practice among women in low- and middle-income countries, 2024.

| Criteria | | A brief description of how the criteria were handled in  the meta-analysis |
| --- | --- | --- |
| **Reporting of background should**  **include** | |  |
| 1 | Problem definition | Page 6: The primary outcome of this review was the magnitude of breast self-examination practices in low- and middle-income countries and the factors associated with breast self-examination practices in low- and middle-income countries. Breast self-examination (BSE) is a general practice a woman can do herself at home that involves looking at and feeling each breast for possible lumps, distortions, or swelling as a preventive measure for breast cancer detection, and who have ever performed BSE (regularly and irregularly) are considered to have practiced BSE. |
|  | Hypothesis statement | Page:4 Early detection of breast cancer plays a crucial role in reducing morbidity and mortality rates. When breast cancer is detected at an early stage, there is a significant chance of achieving a 95% survival rate, Despite the efforts made at low and middle income countries to address the cancer burden in alignment with the United Nations High-Level Meeting on the Control of Non-Communicable Diseases (NCDs) and the Global Action Plan for the Control of NCDs 2013-2020, the primary factors contributing to delayed presentation for breast cancer care in low and middle income countries, particularly in rural areas, are a lack of knowledge and awareness regarding breast self-examination, breast cancer risk factors, early symptoms, perceived vulnerability to breast cancer, and knowledge about early detection methods and treatments are low. |
|  | Description of study outcomes | Page 6: The primary outcome of this review was the magnitude of breast self-examination practices in low- and middle-income countries and the factors associated with breast self-examination practices in low- and middle-income countries. Breast self-examination (BSE) is a general practice a woman can do herself at home that involves looking at and feeling each breast for possible lumps, distortions, or swelling as a preventive measure for breast cancer detection, and who have ever performed BSE (regularly and irregularly) are considered to have practiced BSE. |
|  | Type of exposure or intervention used | Page 5: Breast self-examination practices |
|  | Type of study designs used | Page 5: Umbrella review |
|  | Study population | Page 5: Women |
| **Reporting of the search strategy should include** | |  |
|  | Qualifications of searchers | Page 7: The credentials of the two investigators  BDT, GY, and MA are indicated in the author list. |
|  | Search strategy, including time the period included in the synthesis and keywords | Page 5: The search was conducted from November 26 to 30, 2024. Key words include; breast self-examination, practices, and Low- and middle-income countries. |
|  | Databases and registries searched | Page 5: PubMed, Cochrane Library, research 4 life including Hinari, Google Scholar, CINAHL, and Scopus. |
|  | Search software used, name and version, including special features | Page 7: We did not employ search software. Mendeley  was used to merge retrieved citations and eliminate  Duplications. |
|  | Use of hand searching | Pages 8: We hand-searched bibliographies of retrieved papers for additional references |
|  | List of citations located and those excluded, including justifications | Pages 5: Details of the literature search process are outlined in supplementary file. |
|  | Method of addressing articles published in languages other than English | Page 6: We limited to the studies published in English |
|  | Method of handling abstracts and unpublished studies | Not applicable |
|  | Description of any contact with authors | Not applicable |
| **Reporting of methods should include** | |  |
|  | Description of relevance or appropriateness of studies assembled for assessing the hypothesis to be tested | Pages 6: Detailed inclusion and exclusion criteria were described in the study selection section. |
|  | Rationale for the selection and coding of data | Page 6- 7: Data extracted from each of the studies were relevant to the first author, study characteristics, characteristics of participants, outcome characteristics. |
|  | Assessment of confounding | Not applicable |
|  | Assessment of study quality, including blinding of quality assessors; stratification or regression on possible predictors of study results | Page 7: The quality of the studies was assessed using Assessment of Multiple Systematic Reviews (AMSTAR) checklist scores. |
|  | Assessment of heterogeneity | Page 7: The between studies heterogeneity, which was assessed by Higgins’s I2- Statistics. According to Higgins et al. I2 < 49%, 50–75, and > 75% represents low, moderate, and high levels of heterogeneity, respectively |
|  | Description of statistical methods in sufficient detail to be replicated | Pages 8-12: Description of methods of meta-analyses was detailed in the data synthesis and analysis section. |
|  | Provision of appropriate tables and graphics | We included 1 flow chart, 3 summary tables and 9  Figures |
| **Reporting of results should include** | |  |
|  | Graph summarizing individual study estimates and overall estimate | Figure 2 |
|  | Table giving descriptive information for each study included | Table 1 |
|  | Results of sensitivity testing | Not applicable |
|  | Indication of statistical uncertainty of finding | Pages 12-13: 95% confidence intervals were presented with all summary estimates. |
| **Reporting of discussion should include** | | |
|  | Quantitative assessment of bias | Not applicable since studies were less than 10. |
|  | Justification for exclusion | Pages 6: Papers were excluded based on the exclusion criteria listed. |
|  | Assessment of quality of included studies | AMSTAR checklist scores were used to assess the quality. |
| **Reporting of conclusions should include** | |  |
|  | Consideration of alternative explanations for observed results | Page 24: We discussed the limitations of this study. |
|  | Generalization of the conclusions | Page 1: The findings of this umbrella review indicate that the prevalence of breast self-examination is lower in low- and middle-income countries when compared to developed countries. It is important for stakeholders to take into account the factors identified in this study, and develop strategies to raise awareness and promote the practice of breast self-examination among women in low- and middle-income countries. Implementing breast self-examination effectively can serve as a practical method for early detection of breast cancer, making it crucial to prioritize efforts in this area. |
|  | Guidelines for future research | We recommend future studies on comparing the level of breast self-examination practices between women with breast cancer and women without breast cancer. |
|  | Disclosure of funding source | No funding source |
