# Supplementary material for: Breast self-examination prevalence and determinants in low- and middle-income countries: an umbrella review of systematic reviews and meta-analyses
Source: Front Glob Womens Health. 2026 Apr 1;7:1439187. doi: 10.3389/fgwh.2026.1439187 (PMC13079669; doi:10.3389/fgwh.2026.1439187)
Supplement: Supplementary file 2 [file Supplementaryfile2.docx]

S2 file: Search Strategy in different databases for Prevalence of breast self-examination practice and its determinants among women in low- and middle-income countries, 2024.

| Database | Search | Query | Search results* |
| --- | --- | --- | --- |
| PubMed | #1 | Search: (((((((((((prevalence) OR (proportion)) OR (incidence)) OR (epidemiology)) AND (determinants)) OR (factors)) OR (associated factors))) AND ((breast self-examination))) OR (practices)) OR (factors)) AND (women) OR (low- and middle-income countries) Filters: Meta-Analysis, Systematic Review | 68 |
| Research 4 life | #1 | prevalence and factors of breast self-examination practices in low- and middle-income countries systematic review and meta-analysis | 28 |
| Cochrane library | #1 | Cochrane Reviews matching prevalence in Title Abstract Keyword OR proportion in Title Abstract Keyword AND determinants in Title Abstract Keyword OR factors in Title Abstract Keyword AND breast self-examination practices in low- and middle-income countries in Title Abstract Keyword - (Word variations have been searched) | 8 |
| Other data base | #1 | prevalence and factors of breast self-examination practices in low- and middle-income countries systematic review and meta-analysis | 19 |

**= Date of search: from November 26 to 30, 2024.*
