# Supplementary material for: Breast self-examination prevalence and determinants in low- and middle-income countries: an umbrella review of systematic reviews and meta-analyses
Source: Front Glob Womens Health. 2026 Apr 1;7:1439187. doi: 10.3389/fgwh.2026.1439187 (PMC13079669; doi:10.3389/fgwh.2026.1439187)
Supplement: Supplementary file 3 [file Supplementaryfile3.docx]

S3 file; Methodological quality of the included studies about breast self-examination practice among women in low- and middle-income countries based on the AMSTAR tool, 2024.

| Authors (year) | | Q1 | | Q2 | | | Q3 | | Q4 | | Q5 | Q6 | | | Q7 | | Q8 | Q9 | | | Q10 | Q11 | | | Total |
| --- | --- | --- | --- | --- | --- | --- | --- | --- | --- | --- | --- | --- | --- | --- | --- | --- | --- | --- | --- | --- | --- | --- | --- | --- | --- |
| Gizachew Y et al. (2021) | | yes | | yes | | | yes | | yes | | yes | Yes | | | yes | | yes | yes | | | yes | no | | | 10 |
| Kassie AM et al. (2021) | | yes | | Yes | | | yes | | yes | | yes | No | | | yes | | no | yes | | | yes | yes | | | 9 |
| Mekonnen BD. (2020) | | yes | | yes | | | yes | | yes | | yes | Yes | | | yes | | Yes | yes | | | yes | No | | | 10 |
| Seifu W. (2021) | | yes | | yes | | | yes | | yes | | yes | Yes | | | yes | | yes | yes | | | yes | yes | | | 11 |
| Halim FS. (2023) | | yes | | yes | | | No | | No | | yes | Yes | | | yes | | Yes | Yes | | | Yes | yes | | | 9 |
| Pal A. et al (2021) | | yes | | yes | | | yes | | no | | yes | Yes | | | yes | | yes | yes | | | yes | Yes | | | 10 |
| Ahadinezhad B et al. (2023) | | yes | | Yes | | | yes | | yes | | yes | Yes | | | yes | | no | yes | | | yes | yes | | | 10 |
| Samuel DH et al. (2022) | | yes | | yes | | | yes | | yes | | yes | Yes | | | yes | | no | yes | | | yes | yes | | | 10 |
| Badakhsh M et al. (2018) | | yes | | yes | | | yes | | No | | yes | Yes | | | yes | | yes | yes | | | yes | No | | | 9 |
| Gupta R et al. (2019) | | yes | | yes | yes | | yes | | yes | | | Yes | yes | | yes | | | no | Yes | | | yes | 10 | |  |

*AMSTAR (Assessment of Multiple Systematic Reviews)*

*Q1: A priori design; Q2: Duplicate study selection and data extraction; Q3: Search comprehensiveness; Q4: Inclusion of grey literature; Q5: Included and excluded studies provided; Q6: Characteristics of the included studies provided; Q7: Scientific quality of the primary studies assessed and documented; Q8: Scientific quality of included studies used appropriately in formulating conclusions; Q9: Appropriateness of methods used to combine studies’ findings; Q10: Likelihood of publication bias was assessed; Q11: Conflict of interest – potential sources of support were clearly acknowledged in both the systematic review and the included studies.*
